# Sensitive detection of methylated DNA and methyltransferase activity based on the lighting up of FAM-labeled DNA quenched fluorescence by gold nanoparticles

**DOI:** 10.1039/c9ra01564g

**Published:** 2019-04-16

**Authors:** Mohammad Ali Karimi, Mehdi Dadmehr, Morteza Hosseini, Behnaz Korouzhdehi, Fatemeh Oroojalian

**Affiliations:** Department of Chemistry, Payame Noor University Tehran Iran; Department of Biology, Payame Noor University Tehran Iran mdadmehr@ut.ac.ir; Department of Life Science Engineering, Faculty of New Sciences & Technologies, University of Tehran Tehran Iran; Medical Biomaterials Research Center, Tehran University of Medical Sciences Tehran Iran; Department of Biotechnology, University of Tehran Tehran Iran; Department of Advanced Sciences and Technologies, School of Medicine, North Khorasan University of Medical Sciences Bojnurd Iran; Natural Products and Medicinal Plants Research Center, North Khorasan University of Medical Sciences Bojnurd Iran

## Abstract

DNA methylation of cytosine bases, which is catalyzed by methyltransferase enzymes, involve biochemical processes that contribute to gene expression and gene regulation in cells. Detection of abnormal patterns of both methylated DNA and methyltransferase enzyme activity at early stages could be considered as promising targets for early cancer diagnosis. In the present study, a novel and facile method is introduced for the sensitive detection of the M.SssI methyltransferase (M.SssI MTase) enzyme and methylated DNA based on the fluorescence recovery of FAM-labeled DNA coupled with gold nanoparticles (AuNPs). Thiol-modified probes were functionalized with AuNPs, which brought the FAM fluorophore into the close proximity of the AuNPs. This led to the overlap between the FAM fluorescence emission and AuNPs absorption spectra, introducing a FRET occurrence and causing fluorescence quenching. The hybridization of the probe and its complementary target provided specific CpG sites for M.SssI MTase enzyme activity. The methylation process gradually converted the quenched FAM fluorophore into an emissive fluorophore upon the addition of the MTase enzyme, and the observed fluorescence recovery proved the efficiency of the assay for the detection of MTase enzyme. The fluorescence intensity showed an increasing trend with M.SssI MTase enzyme activity in the range of 1–8 U mL^−1^ with a detection limit of 0.14 U mL^−1^. The addition of methylated ssDNA targets to a ssDNA FAM-labeled probe resulted in a DNA duplex formation, leading to a strong fluorescence signal emission due to the recovery of the fluorophore signal. Conversely, the unmethylated ssDNA target caused no changes in the fluorescence signal. In the presence of methylated DNA targets, the biosensor could specifically recognize it and accordingly trigger the methylated targets through a fluorescence enhancement in the range of 5–100 pM by monitoring the increase in the fluorescence intensity with a detection limit of 2.2 pM. The obtained results showed that the assay could realize the detection of M.SssI MTase and methylated DNA effectively in diluted human serum samples. Human serum conditions showed no significant interference with the assay performance, indicating that the present method has great potential for further application in real samples.

## Abstract 2

A novel method for detection of DNA methylation based on fluorescence recovery of FAM labeled DNA/Au NPs was introduced.

## Graphical abstract

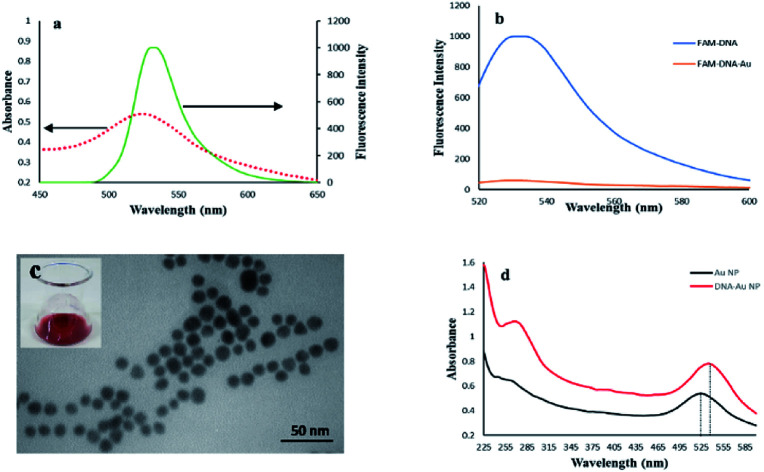

## 1 Introduction

The detection of methylated DNA is the most widely studied epigenetic modification. This process is known as hypermethylation, which consists of the covalent addition of a methyl group from the methyl donor *S*-adenosylmethionine (SAM) to the cytosine within the CpG dinucleotide.^1^ Hypermethylation in the promoter region of tumor suppressor genes have been implicated in downregulated or silenced genes and leads to some cancers.^2–4^ Regulation of DNA methylation is catalyzed by the M.SssI MTase enzyme in the presence of SAM. The abnormal expression and activity of this enzyme is associated with hypermethylation of the promoter region of the tumor suppressor genes. Therefore, the detection of MTase enzyme activity and methylated DNA would be a crucial and efficient predictive biomarker for the early detection of cancers.^5,6^

Recent attempts in biosensor technology could be a determining factor for the development of medical detection systems to reach the maximum assay limit of biomolecules, such as protein and nucleic acid molecules. Previous studies showed that exploiting the advantages of nanomaterials for biosensor fabrication while enhancing their performance improved their detection limit as demonstrated for DNA detection.^7–10^

The conventional methods for the detection of MTase enzyme activity and methylated DNA include bisulfite treatment,^11^ high performance liquid chromatography (HPLC),^12^ electrochemistry,^13–17^ enzyme-linked immunosorbent assay (ELISA),^18^ surface plasmon resonance (SPR) spectroscopy,^19,20^ surface-enhanced Raman scattering (SERS),^21,22^ and use of microfluidic based biosensors^23,24^ and fluorescence based biosensors.^25–28^ Although several attempts have been made for the detection of methylated DNA in recent years, there are still some drawbacks that limit their sensitivity and efficacy in clinical applications.

Among the above-mentioned approaches, the fluorescence based assays (*i.e.*, utilization of fluorophore molecules in a detection system) present remarkable characteristics, including high and specific sensitivity and selectivity with relatively rapid and simple operations, which introduce them as efficient and reliable detection markers. The presence of donor fluorophore and acceptors in specific distances leads to fluorescence resonance energy transfer (FRET) between the fluorescent donors and acceptors. Nowadays, FRET has been considered for the design and fabrication of novel biosensors and detection systems.^29,30^ FAM is a well-known and widespread fluorophore used for the labeling of DNA as a fluorescent marker and can be attached to either the 5′ or 3′ end of oligonucleotides. It can be protonated and has decreased fluorescence below pH 7.

Au nanoparticles (AuNPs) are regarded as one of the most applicable nanoparticles for the detection of biological analytes. Their unique properties include surface attachment to biological elements, such as DNA, proteins, and enzymes. Their absorption properties and high electron conductivity makes them one of the most frequently used biological probes. The absorption and electron transfer capacity properties of AuNPs are dependent on their diameter size, which can be controlled through the parameters of their synthesis. These tunable characteristics introduce them as a very efficient candidate for being an acceptor element in a FRET based detection system for biological studies.^31–33^

**Scheme 1:**
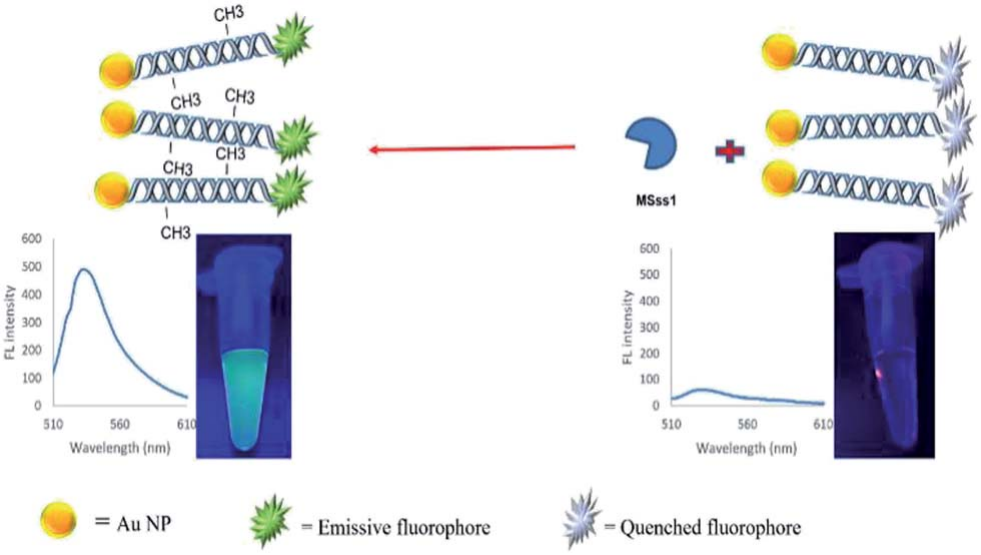
Schematic representation of a FRET based approach for the detection of a methyltransferase enzyme.

**Scheme 2:**
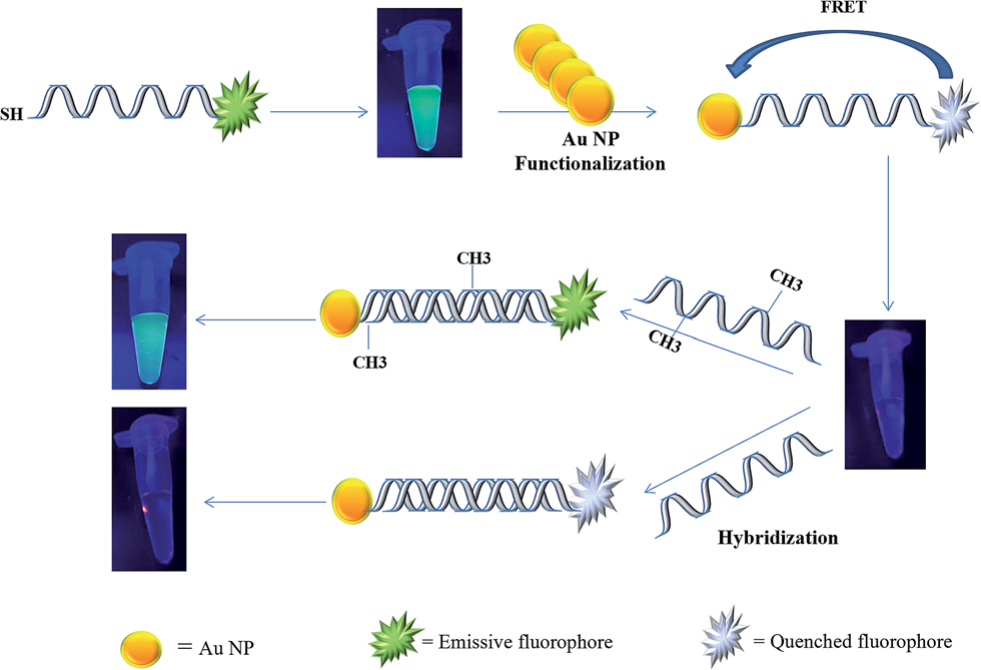
Schematic representation of a FRET based approach for the detection of methylated DNA.

In the present study, a rapid and sensitive FRET based DNA methylation detection method was developed. AuNPs (serving as fluorescence acceptors) were conjugated with a 6-carboxyfluorescein (FAM)-labeled probe employed as the fluorescence donor. Through this process, the proximity of the fluorophore and AuNPs resulted in a FRET occurrence, leading to the fluorescence quenching of the FAM fluorophores. The DNA hybridization of the probe with a complementary target formed a double stranded DNA with a specific inside recognition site for M.SssI MTase enzyme activity. The presence of a 5′-CCGG-3 palindromic site provided the reaction site for initiating the M.SssI MTase activity and DNA methylation process. The activity of the MTase enzyme caused a linear fluorescence recovery of the FAM fluorophore, which showed a relationship with the enzyme concentration and contributed to the enzyme assay (Scheme 1). Meanwhile, for the detection of methylated DNA, the unmethylated and methylated ssDNA targets were designed to hybridize with the functionalized probe during the complementary pairing. In the presence of ssDNA targets, the DNA probes hybridized with the targets and achieved fluorescence recovery for the methylated targets, whereas no changes were observed in the presence of unmethylated targets (Scheme 2). Therefore, it was concluded that the fluorescence recovery of FAM-labeled DNA was closely related to the M.SssI MTase enzyme activity and methylated DNA level. The present novel detection strategy could determine the DNA methylation levels of a specific site of DNA through the sensitive and rapid assay of MTase enzyme and methylated ssDNA targets.

## 2 Experimental details

### 2.1 Reagents and solutions

Tris hydroxymethyl amino methane (Tris-base), chloroauric acid (HAuCl_4_·4H_2_O) and trisodium citrate were purchased from Sigma-Aldrich. Sodium hydroxide (NaOH) and hydrochloric acid (HCl) were obtained from Merck. Ethylenediaminetetraacetic acid (EDTA), dithiothreitol (DTT), phosphate buffer (PB) and sodium dodecyl sulfate (SDS) were purchased from Acros. All chemicals were used without further purification.

The DNA probe and target strands were synthesized according to the CpG sequence sites of the P53 tumor suppressor gene promoter by Shanghai Generay Biotech Co. The oligonucleotide sequences are shown as follows:

Probe sequence (P): 6′-FAM–5′-TCCGGTTCCCGACCCGGACTCCGCAAAAAA-3′-SH

Complementary sequence (S1): 5′-GCGGAGTCCGGGTCGGGAACCGGA-3′

Complementary methylated sequence (S2): 5′-GC(M)GGAGTCC(M) GGGTC(M)GGGAACC(M)GGA-3′

One-base mismatched sequence (S3): 5′-GCGGAGTGCGGGTCGGGAACCGGA-3′

Single base mismatched methylated sequence (S4): 5′-GC(M)GGAGTGC(M)GGGTC(M)GGGAACC(M)GGA-3′

Non-complementary sequence (S5): 5′-AGCCGGCCAAAAGCTGCCAACCGA-3′

All oligonucleotide stock solutions were purified by PAGE, prepared with TE buffer, and kept frozen until used. For the TE buffer preparation, 1 mL of 1 M Tris–HCl (pH 7.5) and 0.2 mL EDTA (0.5 M) were added to deionized water and diluted to a total volume of 100 mL. Human serum samples were obtained from Tehran University of Medical Science, Tehran, Iran and used for the determination of method efficiency in real sample conditions.

### 2.2 Instrumentation

All fluorescence measurements were performed on a Perkin-Elmer LS-55 fluorescence spectrometer with the excitation and emission slits set at 10 nm bandpass. UV-vis spectroscopy was performed by a Specord 250 spectrophotometer (Analytik Jena, Germany). The morphology of the AuNPs was characterized by a transmission electron microscope (TEM) (Zeiss, EM10C, 100 kV, Germany). Deionized water for the preparation of the oligonucleotide stock solution was acquired from a Milli-Q ultrapure water system (Millipore, Z18 MΩ cm).

### 2.3 Synthesis of gold nanoparticles

AuNPs with a mean diameter in the range of 10 ± 3 nm were synthesized by the bottom up method as previously described.^34^ Briefly, 50 mL of an aqueous solution of tetrachloroauric acid (1 mM) was heated to reach the boiling point while being stirred with the magnetic stir bar in a round-bottom flask with reflux modes. Then, 10 mL of trisodium citrate (38.8 mM) was poured into the solution and left to boil for another 10 min. The solution color changed from yellow to purple, and then finally turned wine-red, which was stored in the refrigerator at 4 °C.

### 2.4 Construction of the FAM-oligo-Au probe

Au nanoparticles were functionalized by a fluorophore (FAM) modified 5′-ssDNA probe, which also had a thiol group at the 3′ end. Prior to use, a solution of the reducing agent DTT was added to the lyophilized thiolated DNA probe to reduce the disulfide bonds and prevent the formation of probe dimers. It was then incubated at room temperature for 1 hour (0.1 M DTT, 0.18 M phosphate buffer (PB), pH 8.0). Freshly prepared AuNPs and oligonucleotides probes were added to the solution with phosphate buffer (PB) and sodium dodecyl sulfate (SDS) at 0.01 M and 0.01% concentration, respectively. The ssDNA/AuNP solution was allowed to incubate overnight at room temperature. In order to remove the unbound probes through a washing process, the AuNPs were centrifuged at 14 000 rpm and the supernatant was removed, leaving a red pellet of AuNPs at the bottom of the flask. The precipitated nanoparticles were resuspended in a solution containing 0.01% SDS. This process was repeated 3 times to obtain the FAM-oligo-Au probe.

### 2.5 Detection of M.SssI enzyme activity

In order to assay the M.SssI MTase, the probe strand (FAM-labeled ssDNA) and complementary strand (target 1) were first hybridized at the same concentration in 50 mM Tris–HCl buffer (pH 7), which was incubated at 37 °C for 1 h. Following the incubation, the SAM substrate and buffer solution (10 mM potassium phosphate pH 7.0, 400 mM KCl, 1 mM DTT, 1 mM EDTA, 0.2 mg mL^−1^ BSA and 50% v/v glycerol) with various concentrations of M.SssI MTase (from 1 to 8 U mL^−1^) were added to the solution. The mixture was then incubated at 37 °C for 30 min, and finally the reaction was terminated by the inactivation of the enzymes by further incubating for 20 min at 80 °C. Subsequently, the fluorescence spectra of the newly formed methylated DNA assembly were recorded.

### 2.6 Detection of methylated DNA

To determine the effect of methylated ssDNA target concentrations on the fluorescence recovery efficiency, a fixed amount (1 mg mL^−1^) of the FAM-oligo-Au capture probe was incubated with both unmethylated and methylated ssDNA in the range of 5 pM to 100 pM oligonucleotide concentrations for 2 h to allow hybridization at room temperature. The above mixture would allow the formation of a hybridized sandwich complex structure. After incubation, the fluorescence intensity was measured using a spectrofluorometer. The obtained fluorescence intensities were recorded under the same experimental conditions.

### 2.7 Real sample assay

In order to determine the efficiency of the experiment in a real sample condition, the assay was performed in three different media, namely, (1) buffer (optimum condition) as a control, (2) human serum sample and (3) in the presence of human serum plus interfering DNA. 50 pM of P probes, 50 pM of hybridized P/S3 and 50 pM of hybridized P/S2 were added to each sample, respectively. DNA was then extracted from the above mixtures by a QIAamp DNA Blood Mini Kit (Qiagen) and diluted 2 fold in Tris–HCl buffer. The extracted P/S2 hybrids were analyzed by a spectrofluorometer, while the purified P/S1 hybrids were treated with the M.SssI MTase enzyme for the enzyme assay process.

## 3 Results and discussion

### 3.1 Assay strategy based on FRET between FAM and AuNPs

**Fig. 1:**
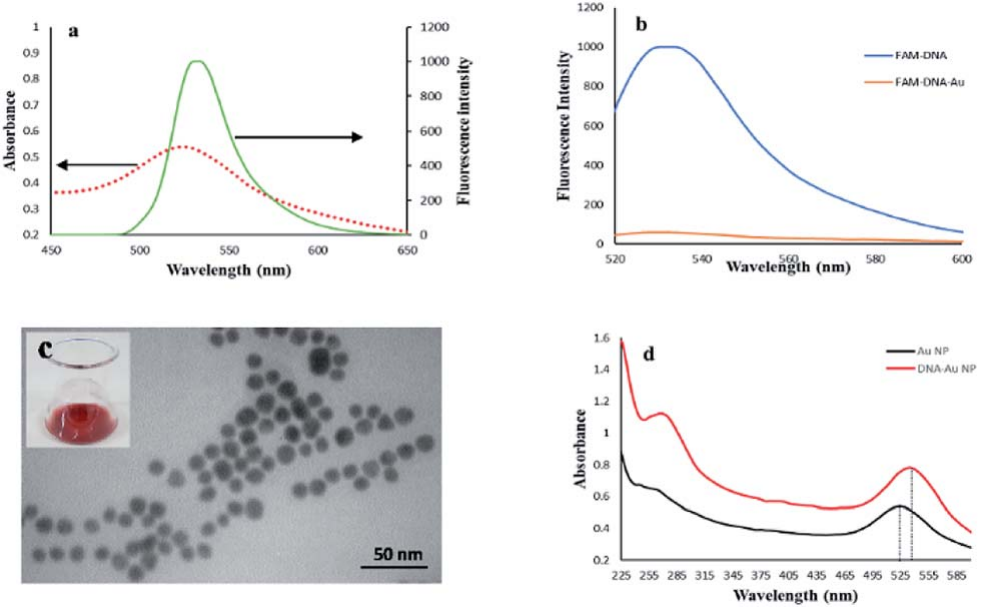
(a) The spectral overlap of the AuNPs absorption spectrum (dashed line) and fluorescence emission spectrum (solid line), (b) fluorescence spectra of FAM-labeled ssDNA before and after functionalization with Au nanoparticles, (c) TEM of AuNPs, inset shows a photograph of the AuNPs solution, (d) UV-vis absorption spectra of the AuNPs and AuNPs-DNA probe. The dotted lines show that the absorption peak of the AuNPs sample experiences a slight red shift from 522 nm to 529 nm.

It was assumed that FAM and AuNPs acting as the donor and acceptor, respectively, would be a favorable pair for FRET. As illustrated in Scheme 1, the FAM-labeled probe that emitted a strong fluorescence was conjugated with AuNPs through the 3′ thiolated end of the probe. The obtained results showed that the fluorescence signal of FAM was quenched efficiently in the presence of certain amounts of AuNPs. This result was due to a significant overlap of the fluorescence emission of FAM with the absorption spectrum of the AuNPs (Fig. 1a). The spectrum of AuNPs showed the absorption peak at 520 nm and its overlap with the FAM functionalized DNA at 540 nm, suggesting that FRET could be occurring between the FAM-labeled DNA (donors) and the AuNPs (acceptors) (Fig. 1b).

The FAM fluorescence quenching resulted from a resonance energy transfer from the FAM to the AuNPs. The fluorescence quenching efficiency reached a fluorescence intensity plateau of 82% when a concentration higher than 1.9 μM of AuNPs was dispersed into the solution. Increasing the AuNPs concentration to more than 1.9 μM did not lead to a decrease in the fluorescence intensity and showed that this concentration is capable of quenching the fluorescence of the FAM-labeled probes. The estimated FRET efficiency or fluorescence quenching efficiency at the same excitation wavelength is defined as:
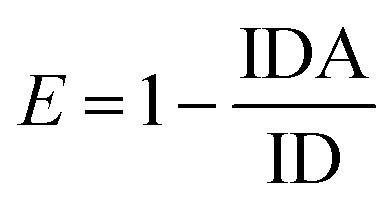
where IDA and ID are the fluorescence intensities of an excited donor fluorophore D in the presence and absence of an acceptor A, respectively. So, the final concentration of AuNPs was determined as 1.9 μM.

### 3.2 Characterization of FAM and AuNPs

The TEM image of the synthesized AuNPs showed that the nanoparticles were well dispersed and exhibited uniform sizes with a diameter ranging from 8 to 12 nm (Fig. 1c), and showed a visible red color when dispersed in ultrapure water (inset of Fig. 1c). The UV-vis spectra of bare AuNPs and FAM-labeled DNA-AuNPs are compared in Fig. 1d. The absorption peak related to the AuNPs-DNA sample at approximately 260 nm was observed, confirming Au functionalization with ssDNA on their surfaces.^35^ Also, as shown in Fig. 1d, the absorption peak of the DNA/AuNPs sample shifted slightly to the right by about 7 nm (from 522 to 529 nm). The observed shift for the DNA/AuNPs sample is attributed to its functionalization with the surface attached DNA probe.^35,36^

### 3.3 Detection of DNA methylation induced by M.SssI MTase

**Fig. 2:**
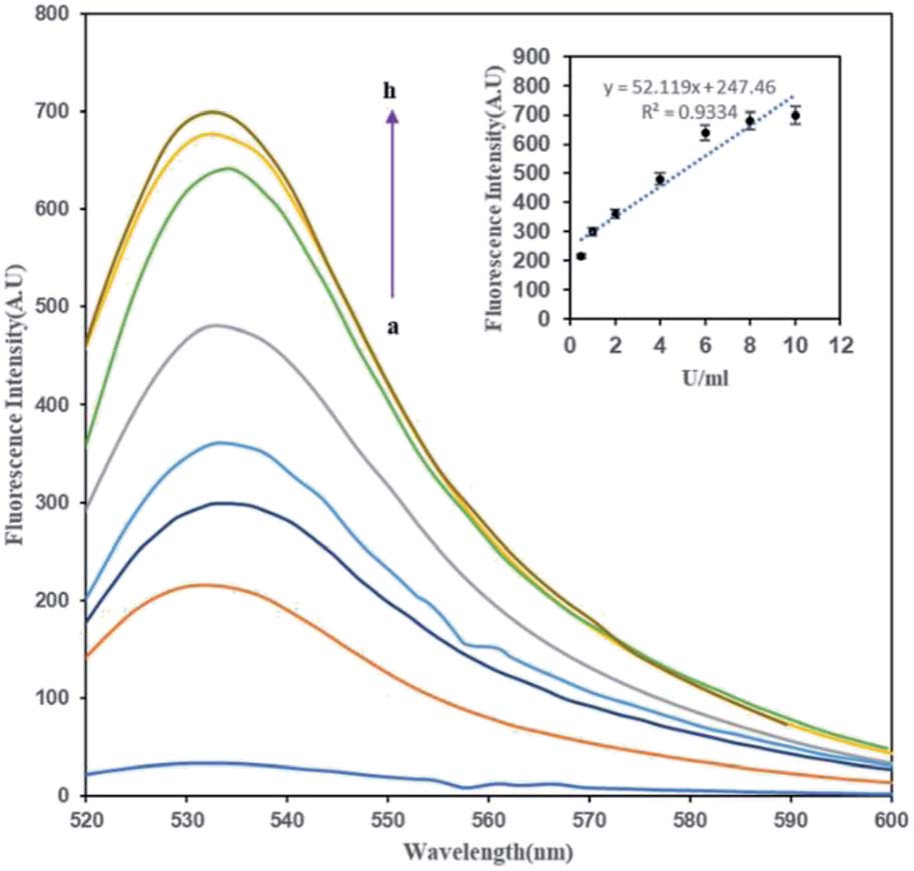
The fluorescence spectra of (1 mg mL^−1^) of FAM-oligo-Au dsDNA probe methylation upon treated with a series of concentration of M.SssI MTase enzyme (a–h: 0, 0.5, 1, 2, 4, 6, 8, 10 U mL^−1^). Inset shows standard curve of relative fluorescence intensity *versus* M.SssI MTase enzyme concentration.

In order to determine the effect of methylation on the FAM-ssDNA-AuNP probes, an unmethylated target was hybridized with the probe to provide –CCGG– sites as a substrate for DNA methylation by the M.SssI MTase enzyme in the presence of SAM. The methylation induction showed the conversion of the quenched FAM fluorophore to a green emitting fluorophore upon DNA methylation. Fig. 2 shows that the fluorescence emission of the treated dsDNA increases with the increase in the M.SssI MTase enzyme concentration from 1 to 8 U mL^−1^. The inset of Fig. 2 clearly shows that the Δ*F* was proportional to the M.SssI MTase concentration from 1 to 8 U mL^−1^ with a correlation coefficient (*R*^2^) of 0.9334. The regression equation was *y* = 52.119*x* + 247.46 and the detection limit of this assay was determined to be 0.14 U mL^−1^.

Previously, it was reported that the activities of enzymes such as proteases and nucleases would be detectable using “switch-on” fluorescent nanoprobes in order to determine their enzyme kinetics or biological activity.^37–40^ Most of the mentioned methods are based on the cleavage function of enzymes on their substrate, which separate the fluorescence donor and acceptor, leading to fluorescence emission from the quenched fluorophore. Also, many reports used oligonucleotide cleavage based methods for MTase enzyme detection.^41,42^ It is worth noting that we reported the cleavage free MTase enzyme detection for the first time. The induction of a methyl group on the DNA structure may contribute to the recovery of the quenched FAM fluorophore through its electron donating activity. Recently, Pongor *et al.* reported that the electron-donor properties of the methyl group possibly enhances the base-stacking interactions that stabilize strand pairing in the stretched state.^43^ They found that in the overstretched state, methylated DNA is longer than the nonmethylated form, suggesting a novel extended *S*-form. In our experience, the above-mentioned changes in the DNA structure and length would have crucial roles in the observed fluorescence recovery of the FAM fluorophore.

Meanwhile, it was illustrated that DNA methylation reduced the FRET efficiency while it was in the nucleosome structure, and indicated that the extent of DNA wrapping or the conformation of DNA was altered upon methylation.^44^ Prior findings indicate that upon the excitation of the sample with polarized light, a favorable dipole orientation (*i.e.*, parallel) of the fluorophore and acceptors and the relative angle between them are determining factors in the FRET efficiency.^45,46^ The FRET orientation factor is called *κ*^2^ and different donor/acceptor conformations can lead to *κ*^2^ values in the 0 ≤ *κ*^2^ ≤ 4 range.^47^ A value of zero for *κ*^2^ means that the process of FRET is forbidden. We assumed that the covalent addition of a methyl group to the DNA could also significantly change the DNA flexibility and conformation. This could lead to different FAM fluorophore and AuNP dipole orientations, resulting in a *κ*^2^ value of zero and subsequently interfering in the FRET occurrence and fluorescence recovery.

### 3.4 Detection of methylated DNA based on FAM/Au NPs FRET system

In order to determine the effect of hybridization on FRET in the present assay, two complementary target DNAs (methylated and unmethylated) were designed. Interestingly, the fluorescence was recovered when the probe was hybridized with the perfectly matched methylated DNA. This effect was not observed for the unmethylated target DNA.

**Fig. 3:**
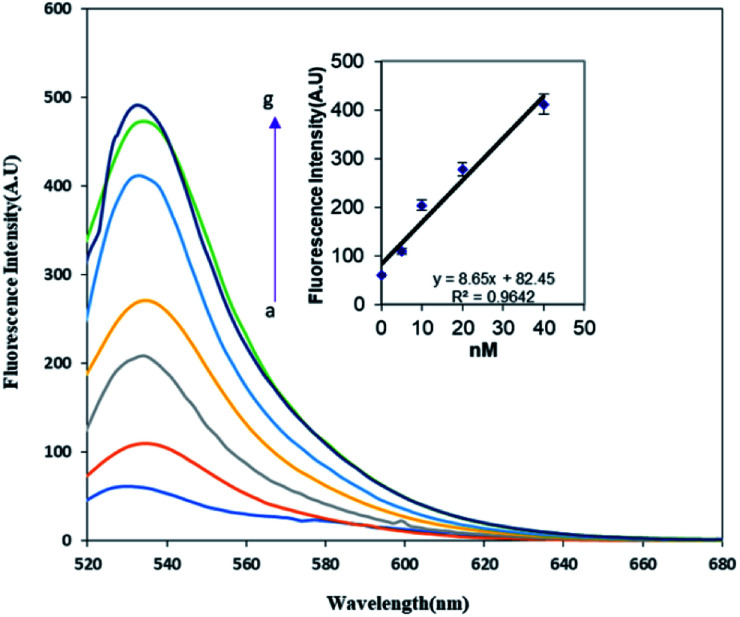
The fluorescence spectra of (1 mg mL^−1^) of FAM-oligo-Au ssDNA probe upon incubation with a series of concentrations of methylated ssDNA targets (a–g: 0, 5 pM, 10 pM, 20 pM, 40 pM, 70 pM, 100 pM). The inset shows a standard curve of the relative fluorescence intensity *versus* the methylated ssDNA target concentration.

As discussed previously, the fluorescence of the FAM labeled oligonucleotide was recovered after the addition of the methylated DNA. The fluorescence intensity pattern of the FAM was monitored relative to the amount of methylated ss-DNA target introduced into the FAM-oligo-AuNPs system. It seems that the presence of a methyl group on the complementary target strand inhibited the energy transfer from FAM to AuNPs and resulted in fluorescence recovery. As depicted in Fig. 3, the fluorescence emission gradually enhanced as the methylated DNA target concentration increased from 5 pM to 100 pM. For the determination of the detection limit of the present FRET system for ssDNA targets, the calibration curve of the detection procedure was plotted in the inset of Fig. 3. The observed results showed that the increase in the methylated ssDNA targets is proportional to the increase in the fluorescence intensity in the linear regression equation *y* = 8.56*x* + 82.45, with a correlation coefficient (*R*^2^) of 0.9642. Finally, the detection limit was determined to be 2.2 pM. Thus, it was concluded that the methylated DNA target can be detected based on the recovered fluorescence intensity and it would be possible to discriminate the methylated DNA from the unmethylated DNA by comparing their fluorescence recovery results.

### 3.5 Application of the assay in a human serum sample

**Fig. 4:**
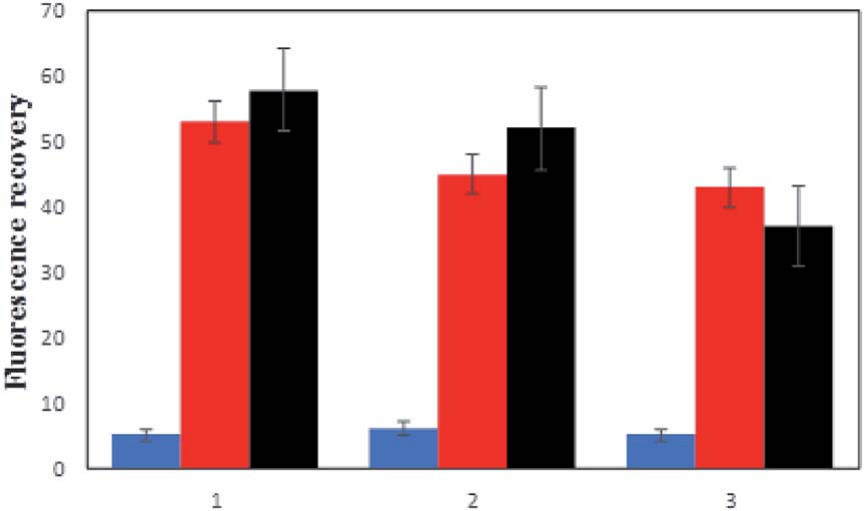
The comparison of fluorescence recovery of P1 (blue columns), P/S2 (red columns) and P/S1 + enzyme (black columns) in a buffer blank sample (series 1), human serum sample (series 2), and human serum sample plus interfering DNA, respectively (series 3).

Determining the applicability of the assay in a real sample is a challenging factor to evaluate the performance of the method. Hence, the human serum as a complex and real substrate was applied in the experiment in the presence of hybridized P/S2 for the detection of methylated DNA and P/S1 strands, as well as for the detection of the MTase enzyme. Meanwhile, non-complementary strands of DNA were used as another interfering material present in human serum as an additional sample. As shown in Fig. 4, the obtained results from the purified human serum DNA (series 2) showed about 84% and 90% fluorescence recovery for the methylated DNA detection method (red columns) and enzyme assays (black columns), respectively. Upon the addition of non-specific DNA (series 3) to the above samples, the fluorescence recovery of the methylated DNA and M.SssI enzyme assay reached 81% and 63%, respectively, which proved the considerable efficiency of the present methods in real samples. Although the presence of non-specific DNA decreased the fluorescence recovery, it did not interfere significantly with the experiment.

As we anticipated, our assay system was able to detect DNA methylation and enzyme activity in a real sample condition. However, further studies are needed in order to fully evaluate and validate the clinical utility of this method.

### 3.6 Sensitivity of experiment

**Fig. 5:**
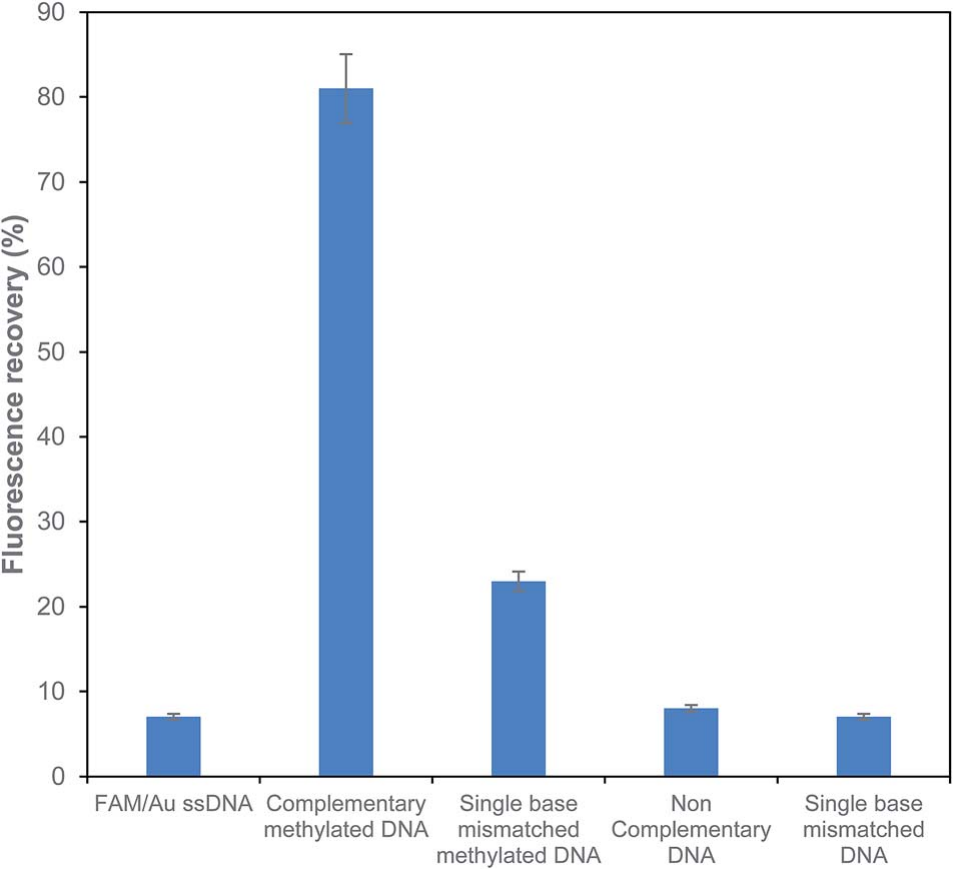
Sensitivity analysis of the fluorescence recovery response for different types of ssDNA targets.

To explore the sensitivity for the detection of methylated DNA, the effects of a complementary methylated sequence (P/S2), a single base mismatched sequence (P/S3), a single base mismatched methylated sequence (P/S4) and a non-complementary sequence (P/S5) were examined at the same concentration for comparison. As depicted in Fig. 5, a comparison of the above-formed oligonucleotides was performed and the obtained results showed that the fluorescence intensity of FAM just recovered with a mismatched target at a very low efficiency of around 29%. The fluorescence recovery was not observed with the addition of non-complementary and single base unmethylated targets. It is worth noting that the presently described FRET based assay has an efficient capacity for the determination of a methylated sequence. The obtained results also suggested that there was no obvious influence for the detection of a methylated DNA in the presence of the potential interfering sequences at the same concentrations.

## 4 Conclusion

Here, we exploited a new strategy, in which the FAM fluorescence donor could de-excite through a methylation process for the detection of DNA methylation. To construct the biosensor, FAM fluorophore labeled probes containing the thiol modified tails were designed. After conjugation with AuNPs, the complementary unmethylated targets were used as targets to form a dsDNA duplex and provide a substrate for the M.SssI MTase enzyme activity assay. The MTase enzyme activity resulted in the fluorescence recovery upon an increase in the enzyme concentration. A similar behavior was also observed upon the addition of methylated ssDNA targets. In our assumption, the DNA orientation after DNA methylation may cause the *k*^2^ value to approach zero, forbidding the FRET occurrence and resulting in the fluorescence recovery. This FRET based system provided an efficient and simple method rather than other conventional multistep methods for the detection of DNA methylation. The overall results clearly illustrated that this introduced novel FRET-based strategy was sensitive enough with great potential to detect MTase activity and determine the presence of the methylated DNA. The performance of this experiment in human serum had no significant effect on the assay performance, holding great potential for further clinical application.

## Conflicts of interest

There are no conflicts to declare.

## Supplementary Material
